# Surgery for herniated lumbar disc in private vs public hospitals: A pragmatic comparative effectiveness study

**DOI:** 10.1007/s00701-019-04195-7

**Published:** 2020-01-04

**Authors:** Mattis A. Madsbu, Øyvind Salvesen, Sven M. Carlsen, Steinar Westin, Kristian Onarheim, Øystein P. Nygaard, Tore K. Solberg, Sasha Gulati

**Affiliations:** 1 Department of Neurosurgery, St. Olavs University Hospital, Trondheim, Norway; 2 Department of Neuroscience, Norwegian University of Science and Technology (NTNU), Trondheim, Norway; 3 Department of Public Health and Nursing, Norwegian University of Science and Technology (NTNU), Trondheim, Norway; 4 Department of Clinical and Molecular Medicine, Faculty of Medicine and Health Sciences, Norwegian University of Science and Technology (NTNU), Trondheim, Norway; 5 Department of Endocrinology, St Olavs Hospital, Trondheim, Norway; 6 Central Norway Regional Health Authority, Stjørda, Norway; 7 National Advisory Unit on Spinal Surgery, St. Olavs University Hospital, Trondheim, Norway; 8 The Norwegian National Registry for Spine Surgery, University Hospital of Northern Norway (UNN), Tromsø, Norway; 9 Department of Neurosurgery, University Hospital of Northern Norway (UNN), Tromsø, Norway; 10 Institute of Clinical Medicine, The Arctic University of Norway (UIT), Tromsø, Norway

**Keywords:** Intervertebral disc displacement, Neurosurgery, Orthopedics, Public health, Sciatica

## Abstract

**Background:**

There is limited evidence on the comparative performance of private and public healthcare. Our aim was to compare outcomes following surgery for lumbar disc herniation (LDH) in private versus public hospitals.

**Methods:**

Data were obtained from the Norwegian registry for spine surgery. Primary outcome was change in Oswestry disability index (ODI) 1 year after surgery. Secondary endpoints were quality of life (EuroQol EQ-5D), back and leg pain, complications, and duration of surgery and hospital stays.

**Results:**

Among 5221 patients, 1728 in the private group and 3493 in the public group, 3624 (69.4%) completed 1-year follow-up. In the private group, mean improvement in ODI was 28.8 points vs 32.3 points in the public group (mean difference − 3.5, 95% CI − 5.0 to − 1.9; *P* for equivalence < 0.001). Equivalence was confirmed in a propensity-matched cohort and following mixed linear model analyses. There were differences in mean change between the groups for EQ-5D (mean difference − 0.05, 95% CI − 0.08 to − 0.02; *P* = 0.002) and back pain (mean difference − 0.2, 95% CI − 0.2, − 0.4 to − 0.004; *P* = 0.046), but after propensity matching, the groups did not differ. No difference was found between the two groups for leg pain. Complication rates was lower in the private group (4.5% vs 7.2%; *P* < 0.001), but after propensity matching, there was no difference. Patients operated in private clinics had shorter duration of surgery (48.4 vs 61.8 min) and hospital stay (0.7 vs 2.2 days).

**Conclusion:**

At 1 year, the effectiveness of surgery for LDH was equivalent in private and public hospitals.

## Introduction

Public health care is usually provided by the government through national healthcare systems, whereas private health care is often provided as “for profit” services. Ideological debates whether countries should strengthen public versus private healthcare services are common. In times of economic recession with constraints on government budgets, disputes between the proponents of private and public health care systems tend to escalate. Discussions about resource allocation between private and public health providers should be evidence based, and focused on clinical effectiveness and costs. There is currently limited and only poor-quality evidence regarding the comparative performance of the two health care systems [2]. In order to achieve a more informed policy, there is an urgent need for robust evidence by comparing the quality and effectiveness of the health care provided through both systems. Degenerative lumbar spine disorders are a leading cause of activity limitation and work absence throughout much of the world, and places an enormous economic burden on the whole society ranging from individuals, families, communities, industry, and all the way to governments. The most common reason for spine surgery is persisting or intolerable pain due to sciatica caused by lumbar disc herniation [4, 19]. In many countries, surgical management of degenerative lumbar spine disorders is provided by both public and private hospitals, providing a unique opportunity to compare the two health care provider systems. The aim of this study was to compare patient-reported outcomes following surgery for lumbar disc herniation (LDH) because of sciatica in public versus private hospitals.

## Methods

Reporting is consistent with the strengthening the reporting of observational studies in epidemiology (STROBE) statement. [25]

### Ethical approval

This study was approved by the regional committee for medical research in central Norway (ID2016/840) and all participants provided written informed consent. The Data Inspectorate of Norway approved the registry protocol.

### Study population

Norway has a public healthcare system with equal distribution of resources and uniform training and licensing of healthcare staff. The population is relatively homogenous and stable. Patients utilizing the public healthcare system are usually treated at the hospital serving their residential address, limiting referral bias. Surgery for LDH is provided free of cost to patients in the public healthcare system. In private hospitals, expenses of surgery (approximately USD 5400 for single-level lumbar microdiscectomy) are covered by the patients themselves or their insurance providers. Many of the patients treated at private hospitals have private health insurance paid for by their employers. During the study period, some of the private hospitals had government funding through contracts with the public regional health authorities.

We collected data through the Norwegian Registry for Spine Surgery (NORspine), a comprehensive registry for quality control and research [17]. In total, 37 of 40 centers performing lumbar spine surgery in Norway reported to NORspine during the study period. According to the Norwegian Directorate of Health, approximately 63% of all patients who underwent lumbar spine surgery in Norway during the study period were included in NORspine [17]. In general, the departments that participated in this study had the same preferred surgical strategy for LDH without radiological instability. Surgery was performed by ipsilateral paravertebral muscle retraction and removal of the disc herniation under microscope magnification by a unilateral transflaval approach [1]. Participation in the NORspine register was not mandatory for providers or patients, and it was not required for a patient to gain access to health care or for a provider to be eligible for payment. Follow-up time from the date of the operation was 1 year. Patients included in the study were treated at hospitals reporting at least 50 patients to NORspine during the study period.

We screened patients who underwent surgery between January 2007 and May 2014 for eligibility. Follow-up time from the date of the operation was 1 year.

We considered patients eligible for the study if they had a diagnosis of symptomatic paramedian lumbar disc herniation, surgery was performed as a single-level lumbar microdiscectomy, and their data were included in the NORspine registry. Patients were excluded who had undergone previous surgery of the lumbar spine, undergone fusion and/or open laminectomy as a surgical approach, or had associated spinal conditions (degenerative spondylolisthesis and/or scoliosis).

### User involvement

The Norwegian Back Pain Association (Ryggforeningen) reviewed the study protocol and provided feedback concerning study design and outcome measures.

### Outcome measures

The primary outcome was change in disease-specific functional outcome between baseline and 12-month-follow-up measured with version 2.1 of the Oswestry disability index [5], which has been translated into Norwegian and tested for psychometric properties [8]. The Oswestry disability index questionnaire quantifies disability for degenerative conditions of the lumbar spine. It covers intensity of pain, ability to lift, ability to care for oneself, ability to walk, ability to sit, sexual function, ability to stand, social life, sleep quality, and ability to travel. For each topic, there are six statements describing potential scenarios, and patients select the one that most closely resembles their situation. The index is scored from 0 to 100. Zero means no disability and 100 reflects maximum disability.

Secondary outcome measures were changes between baseline and 12-month follow-up in generic health-related quality of life, measured with the generic Euro-Qol-5D (EQ-5D), and intensity of back pain and leg pain. The Norwegian version of EQ-5D has shown good psychometric properties [20]. Intensity of pain was graded in two separate 0–10 numerical rating scales (NRS) for back pain and leg pain where 0 equals no pain and 10 represents the worst conceivable pain. The NRS pain scales and ODI have shown good validity and are frequently used in research on back pain [8, 20]. We also compared duration of procedures, length of hospital stays, reoperation at the index level within 3 months of surgery, and surgical complication rates. Surgeons provided the following complications and adverse events to NORspine: intraoperative hemorrhage blood replacement or postoperative hematoma, unintentional durotomy, cardiovascular complications, respiratory complications, anaphylactic reactions, and wrong level for surgery. Patients reported the following complications if occurring within 3 months of surgery: wound infection, urinary tract infection, micturition problems, pneumonia, pulmonary embolism, and deep vein thrombosis.

### Data collection by NORspine

On admission for surgery, the patients completed the baseline questionnaire, which included questions about demographics and lifestyle issues in addition to the outcome measures. During the hospital stay, using a standard registration form, the surgeon recorded data concerning diagnosis, previous lumbar spine surgery, comorbidity, American Society of Anesthesiologists (ASA) grade, image findings, and surgical approach and procedure. A questionnaire was distributed to patients by regular mail at 3 months and 1 year after surgery, completed at home by the patients, and returned in the same way. The patients who did not respond received one reminder with a new copy of the questionnaire. The patients completed baseline questionnaire data and postal follow-up questionnaires without any assistance from the surgeon or other staff from the treating hospital.

### Statistical analysis

Statistical analyses were performed with SPSS version 23.0 (IBM Corporation, Chicago, IL, USA) and Software R [23]. The size of the study was based on a null hypothesis on non-equivalence and an alternative hypothesis of equivalence. If the population effect of treatment on changes in ODI was eight points or less, treatments were considered equivalent for effectiveness [3, 12, 18]. The sample size calculation relates to a two one-sided test for equivalence, with a significance level of 2.5%. We computed the *P* values for equivalence as 1 minus the maximum confidence level at which the confidence interval is contained in (− 8 to 8) divided by 2 giving the *P* values of the two one-sided test for equivalence. This applied to both the complete case analysis and the mixed linear model analysis in both the aggregate cohort and the propensity-matched cohort. Assuming a correlation of 0.5 between baseline and follow-up measurements and a standard deviation of 18 for the individual measurements, this study has a 90% power, with 340 patients in each group.

For statistical comparison tests, we defined the significance level defined as *P* ≤ 0.05 on the basis of a two-sided hypothesis test with no adjustments made for multiple comparisons. For the primary outcome and one secondary outcome (EQ-5D), a statistician (ØS) blinded to treatment provider performed both a complete case analysis and a full information analysis using mixed linear models. Central tendencies are presented as means when normally distributed and as medians when skewed. We used the Chi square test for categorical variables. Baseline- and 1-year scores were compared with paired-samples *t* test. Mean change scores between the groups were analyzed with independent-samples *t* test for complete cases and mixed linear models on all available data. For mixed linear models, the combination of patients operated in private or public clinics and time was taken as fixed effect and participant ID specified as random effect. A multiple linear regression model was applied to assess the relationship between the change in ODI score at 1 year (dependent variable) and private or public treatment, controlling for potential confounders. The selection of predictors included was based on their clinical importance and association with the dependent variable [6, 10, 16].

To achieve equality, we eliminate as many as possible confounding factors and provide best possible balance between the two groups; we generated propensity scores using logistic regression and adjusting for baseline covariates that could influence clinical outcomes, including age, sex, smoking, college education, partner, year of operation, BMI, ASA grade > 2, relevant comorbidity, emergency operation, duration of sciatica > 1 year, and preoperative ODI score. This was to achieve the closest approximate to a randomized clinical trial.

All covariates were entered into a logistic regression analysis, and we fitted a maximum likelihood model based on these covariates as predictors of private versus public treatment. The coefficients for these predictors of private versus public treatment was used to calculate a propensity score of 0 to 1 for each patient. Based on the calculated propensity scores, two evenly matched groups were formed for private and public treatment using a matching algorithm with the common caliper set at 0.010. This dataset is referred to as the “propensity-matched cohort”. We have analyzed continuous variables using a related sample two-tailed *t* test for data with a normal distribution and continuous data exhibiting a skewed distribution using the Wilcoxon signed rank test for matched pairs. We used the McNemar’s test for correlated proportions to compare discrete variables. We handled missing data with mixed linear models and did not perform multiple imputations. This strategy was in line with studies showing that it is not necessary to handle missing data using multiple imputations before performing a mixed model analysis on longitudinal data [15, 24].

## Results

### Baseline characteristics

A total of 5221 patients were included, 1728 operated in private hospitals and 3493 in public hospitals. Participants underwent surgery at 24 orthopedic or neurosurgical departments in 22 hospitals in Norway, 14 public and 8 private. Baseline characteristics were stratified by type of treatment center and matching (Table 1). In the aggregate cohort, there were significant differences between the two groups for baseline characteristics including age, sex, educational level, body mass index, tobacco use, comorbidity, American Society of Anesthesiologists grade, mean baseline Oswestry disability index score, mean baseline EQ-5D, mean NRS back and leg pain, and number of emergency surgery procedures. After propensity score matching (1281 pairs), these differences in baseline characteristics disappeared. The loss to follow-up rate in the aggregate cohort at 1 year was 30.9% (*n* = 533) in the private group and 30.5% (*n* = 1064) in the public group (*P* = 0.77). In the propensity-matched cohort, the loss to follow-up rate in the private group was 32.5% (*n* = 416) and 31.2% (*n* = 400) for the public group (*P* = 0.52). There were no differences between non-responders and responders at 1 year for preoperative ODI, preoperative back pain, preoperative leg pain, preoperative EQ-5D, comorbidity, or ASA grade. However, there were differences between non-responders and responders in age (40.2 vs 45.6; *P* < 0.001), BMI (27.0 vs 26.5; *P* = 0.05), female sex (36.9% vs 42.5%; *P* < 0.001), college education (33.8% vs 41.4%; *P* < 0.001), and tobacco smoking (34.6% vs 26.4%; *P* < 0.001).

**Table 1:** Personal characteristics, coexisting illnesses, and measures of health status for both treatment groups in aggregate and propensity-matched cohorts. Values are numbers (percentages) unless stated otherwise

Variables	Aggregate cohort		*P* value	Propensity-matched cohort		*P* value
	Private hospitals (*n* = 1728)	Public hospitals (*n* = 3493)		Private hospitals (*n* = 1281)	Public hospitals (*n* = 1281)	
Age (years)	43.3	44.3	0.01	42.9	42.9	0.96
Female sex	622 (36%)	1506 (4.1%)	< 0.001	253 (31.8%)	255 (32%)	0.97
Life partner/married	1313 (76.5%)	2605 (75.3%)	0.17	228 (75.7%)	225 (75.5%)	0.93
Attended college	783 (45.7%)	1243 (35.8%)	< 0.001	200 (26.8%)v	198 (26.6%)	0.96
Body mass index (BMI)	26.3	26.7	0.001	26.4	26.4	0.84
Current smoker	443 (25.9%)	1050 (30.4%)	0.001	228 (24.2%)	219 (23.4%)	0.71
Comorbidity	247 (14.3%)	823 (23.6%)	< 0.001	134 (12.5%)	146 (13.5%)	0.51
ASA > 2	29 (1.7%)	168 (4.9%)	< 0.001	21 (1.7%)	13 (1.0%)	0.23
Preoperative ODI	40.9	47.5	< 0.001	41.3	41.4	0.91
Preoperative EQ-5D	0.34	0.25	< 0.001	0.4	0.3	0.80
Preoperative back pain	5.6	6.4	< 0.001	5.7	5.8	0.14
Preoperative leg pain	6.5	7.0	< 0.001	6.5	6.5	0.60
Emergency surgery	18 (1.0%)	1011 (29.1%)	< 0.001	11 (0.8%)	11 (0.8%)	–

### Primary outcome

**Fig. 1:**
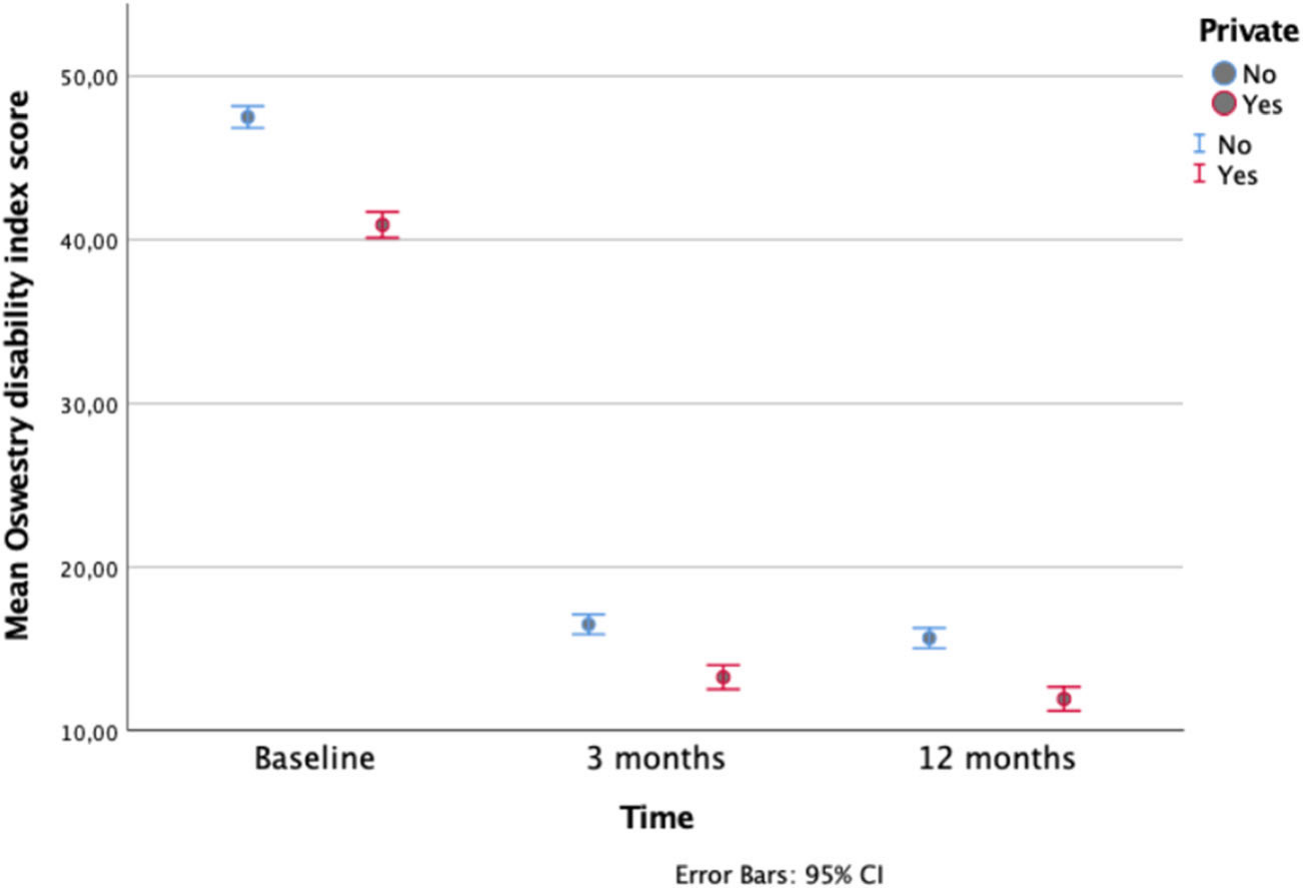
Change in Oswestry disability index score after microdiscectomy for lumbar disc herniation in aggregate cohort during 1-year follow-up for patients operated in private versus public hospitals

**Fig. 2:**
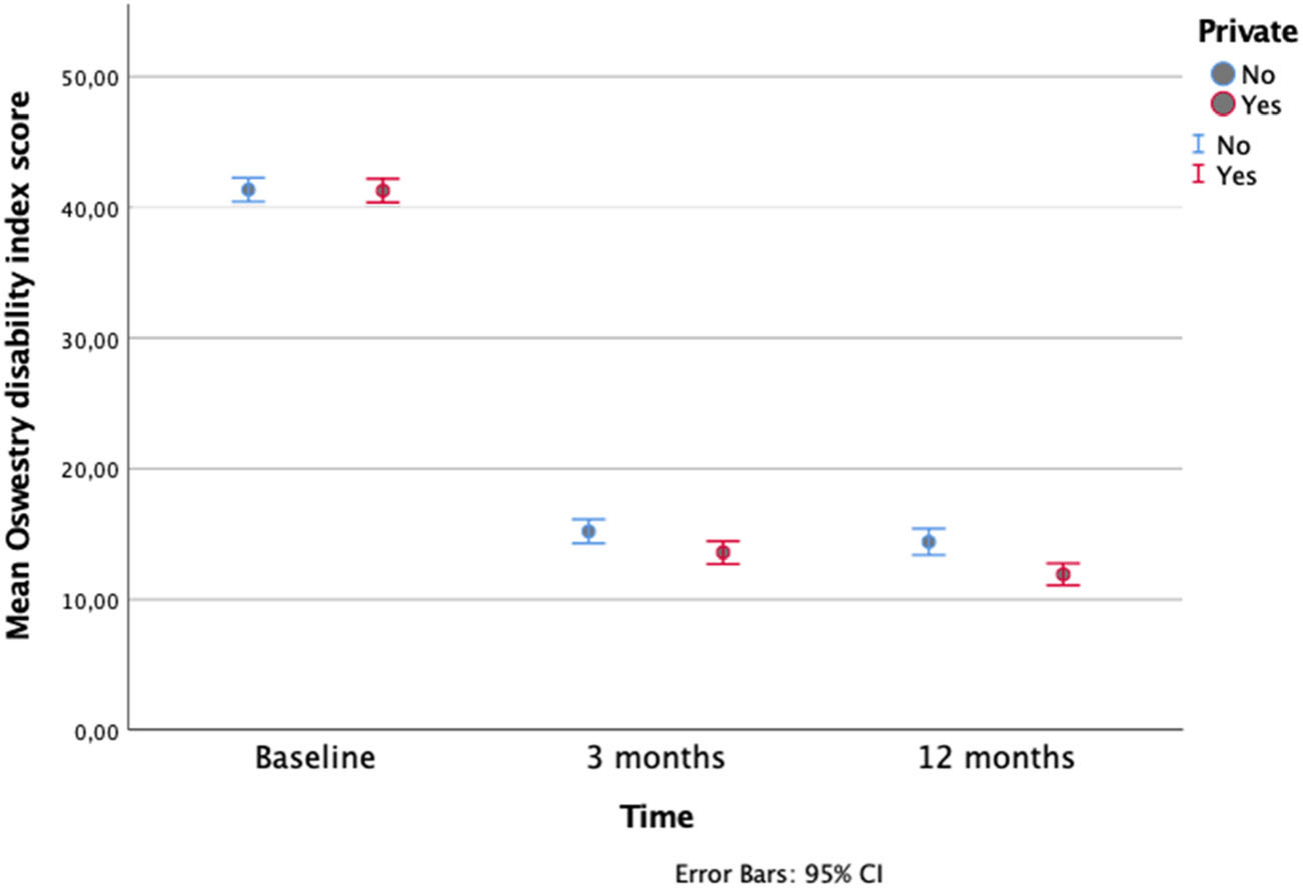
Change in Oswestry disability index score after microdiscectomy for lumbar disc herniation in propensity-matched cohort during 1-year follow-up for patients operated in private versus public hospitals

Complete case analyses and mixed linear model analyses for outcomes in both the aggregate and propensity-matched cohorts at 1 year are presented in Table 2. Figures 1 and 2 show the primary outcomes in the aggregate and propensity-matched cohorts during 1 year of follow-up. For the private and public group combined, the mean improvement in ODI was 31.1 (95% CI 30.4 to 31.9; *P* < 0.001) in the aggregate cohort and 28.4 (95% CI 27.5 to 29.3; *P* < 0.001) in the propensity-matched cohort. For the private group in the aggregate cohort, the improvement in ODI was 28.8 points vs 32.3 in the public group (mean difference 95% CI − 5.0 to − 1.9; *P* for equivalence < 0.001). Equivalence was confirmed in the propensity-matched cohort (mean difference 2.0, 95% CI − 0.25 to 4.3; *P* < 0.001 for equivalence).

**Table 2:** Complete case analyses and mixed linear model analysis for outcomes at 1 year in patients operated in private or public hospitals for lumbar disc herniation

Outcomes	Complete case analysis (*N* = 3624)						Difference in mean change between groups (95% CI)	*P* for equivalence		Mixed model analysis						Difference in mean change between groups (95% CI)	*P* for equivalence
	Private hospitals (*N* = 1195)			Public hospitals (*N* = 2429)						Private hospitals			Public hospitals				
	Baseline	1 year	Mean change	Baseline	1 year	Mean change				Baseline	1 year	Mean change	Baseline	1 year	Mean change		
Aggregate cohort																	
ODI	40.9	11.9	28.8	47.5	15.7	32.3	− 3.5 − 5.0, − 1.9)	< 0.001		41.0	12.0	29.0	47.5	15.6	31.9	− 2.9 (− 4.3, − 1.5)	< 0.001
EQ-5D	0.34	0.80	0.45	0.25	0.74	0.50	− 0.05 (− 0.08, − 0.02)	–		0.34	0.80	0.46	0.24	0.74	0.50	− 0.04 (− 0.07, − 0.01)	–
Back pain	5.6	2.3	3.3	6.4	2.8	3.5	− 0.2 (− 0.4, − 0.004)	–		5.6	2.3	3.3	6.4	2.8	3.6	− 0.2 (− 0.4, − 0.01)	–
Leg pain	6.5	1.8	4.8	7.01	2.3	4.8	− 0.01 (− 0.2, 0.2)	–		6.5	1.8	4.7	7.01	2.3	4.8	− 0.03 (− 0.2, 0.2)	–
Matched cohort	Private hospitals (*N* = 865)			Public hospitals (*N* = 881)													
ODI	41.3	11.4	29.0	41.4	14.0	27.0	2.0 (− 0.3, 4.3)	< 0.001		41.3	12.0	29.3	41.4	14.4	27.0	2.3 (0.6, 4.0)	< 0.001
EQ-5D	0.35	0.82	0.46	0.34	0.78	0.42	0.04 ( − 0.01, 0.1)	–		0.35	0.81	0.46	0.34	0.76	0.42	0.04 (− 0.01, 0.1)	–
Back pain	5.7	2.2	3.4	5.8	2.6	3.2	0.3 (− 0.1, 0.6)	–		5.7	2.3	3.4	5.8	2.7	3.2	0.3 (0.0, 0.5)	–
Leg pain	6.5	1.8	4.8	6.5	2.0	4.5	0.3 (− 0.04, 0.7)	–		6.5	1.8	4.8	6.5	2.1	4.4	0.4 (0.1, 0.6)	–

### Secondary outcomes

There was a small difference in mean change between the groups in the aggregate cohort in favor of the public group for EQ-5D (0.25 vs 0.50, mean difference − 0.05, 95% CI − 0.08 to − 0.02; *P* = 0.002) and back pain (3.3 vs 3.5, mean difference − 0.2, 95% CI − 0.22, − 0.44 to − 0.004; *P* = 0.046). After propensity matching, the groups did not differ (0.46 vs 0.42 for EQ-5D; *P* = 0.13) and (3.4 vs 3.2 for back pain; *P* = 0.16). There was no difference between the two groups for leg pain. Duration of surgical procedures and length of hospital stays were lower in the private group compared to the public group (for the matched cohorts 47.9 vs 57.8 min; *P* < 0.001 and 0.7 vs 2.0 days; *P* < 0.001), as shown in Table 3. For the aggregate cohort, there was a significantly higher number of patients operated in a public hospital that experienced both perioperative complications and postoperative complications within 3 months (2.9% vs 1.4%; *P* = 0.001) and (6.5% vs 4.5%; *P* = 0.020), respectively. In the propensity-matched cohort, there were no differences in either perioperative or postoperative complications within 3 months between the two groups.

**Table 3:** Operation time, complications, and events. Values are numbers (percentages) of participants unless stated otherwise

Variables	Aggregate cohort		*P* value	Propensity-matched cohort		*P* value
	Private hospitals (*n* = 1728)	Public hospitals (*n* = 3493)		Private hospitals (*n* = 1281)	Public hospitals (*n* = 1281)	
Operation time (min)	48.4	61.8	< 0.001	47.9	57.8	< 0.001
Days in hospital (no.)	0.7	2.2	< 0.001	0.7	2.0	< 0.001
Patients with complications (no.)	78 (4.5%)	250 (7.2%)	< 0.001	56 (4.7%)	76 (6.2%)	0.10
Perioperative complications (no.)	24 (1.4%)	100 (2.9%)	0.001	18 (1.4%)	30 (2.4%)	0.11
Dural tear or CSF leak	12 (0.7%)	61 (1.7%)	0.002	9 (0.7%)	21 (1.7%)	0.04
Nerve injury	6 (0.3%)	7 (0.2%)	0.38	4 (0.3%)	2 (0.2%)	0.69
Blood replacement or postoperative hematoma	8 (0.5%)	10 (0.3%)	0.32	6 (0.5%)	1 (0.1%)	0.13
Cardiovascular compl.	–	5 (0.1%)	0.18	–	–	–
Respiratory compl.	1 (0.1%)	1 (−)	0.55	1 (0.1%)	–	–
Anaphylactic reaction	3 (0.2%)	3 (0.1%)	0.40	2 (0.2%)	1 (0.1%)	1.0
Wrong level surgery	–	8 (0.2%)	0.06	–	1 (0.1%)	–
Complications within 3 months (no.)	55 (4.5%)	157 (6.5%)	0.02	24 (3.9%)	33 (5.3%)	0.29
Wound infection	41 (3.4%)	62 (2.6%)	0.17	18 (2.9%)	18 (2.9%)	1.0
Urinaty tract infection	3 (0.2%)	47 (1.9%)	< 0.001	2 (0.3%)	8 (1.2%)	0.11
Micturition problems	10 (0.8%)	47 (1.9%)	0.01	4 (0.6%)	7 (1.1%)	0.55
Pneumonia	3 (0.2%)	10 (0.4%)	0.56	2 (0.3%)	4 (0.6%)	0.69
Pulmonary embolism	1 (0.1%)	0 (−)	0.33	–	–	–
Deep venous thrombosis	–	2 (0.1%)	1.0	–	–	–
Reoperated within 90 days no. (%)	20 (1.2%)	44 (1.3%)	0.79	10 (0.8%)	10 (0.8%)	1.0

## Discussion

To our knowledge, this is the first study to compare patient-reported outcomes between private and public hospitals. Despite differences in patients’ baseline characteristics that may influence treatment outcomes, the effectiveness of lumbar microdiscectomy was equivalent in public and private hospitals in this registry-based multicenter observational study. This finding was consistent in both unmatched and propensity-matched populations.

Duration of surgery was shorter in private hospitals. This may in part be explained by the surgical team’s experience. Unlike private hospitals, most public hospitals are teaching institutions where surgical residents and operating room staff are learning the procedure and working under guidance and supervision. It is also possible that surgical units specializing in fewer procedures, microdiscectomy being one of them, are prone to develop a more efficient take on the surgical technique and logistics, resulting in shorter operation time. They also avoid the burden of having a readiness for acute interventions that in the public part of this cohort was as high as 29.1%.

Longer hospital stays in public hospitals could partly be explained by the fact that public hospitals only received full reimbursement from Norwegian health authorities if the patients spent the first night following surgery in hospital. It is also rather common that patients originally referred to private hospitals are rejected when having comorbidities or other factors that may negatively influence outcomes and logistics [9]. Lumbar microdiscectomy seems to be a safe surgical procedure with few serious complications. In the aggregate cohort, there were slightly more perioperative complications within 3 months in the public group compared to the private group. These differences disappeared following propensity matching, supporting the evidence that “healthier” patients are treated in private hospitals [9].

Unlike private hospitals, most public hospitals are teaching institutions where surgical residents and operating room staff are learning the procedure and working under guidance and supervision. Our results are in line with a study showing that surgical treatment of LDH at public hospitals with dedicated training programs does not lead to inferior patient care [22].

Economic and social differences between patients and access to healthcare are not as big of a challenge in Norway compared to other parts of the world. This could in part explain our equivalent results. However, a systematic review from low- and middle-income countries showed that the private sector was not superior to the public when considering medical efficiency [2].

Considering the equivalence of surgical results between the two health care providers in our nationwide patient sample, one could argue that there is a widespread access to high quality surgical management of LDH in both private and public health care systems. The dilemma each patient should consider is then not the effectiveness and quality of the health care, but rather their own economical capacity and possible waiting time for surgery. The role of private health insurance also comes under scrutiny in a country where the public health care system is well functioning and provides all emergency and complex in-house medical treatment [11].

### Study strengths and limitations

The major strength of this study is our use of propensity-matched groups to minimize confounding factors. Other strengths include the large sample size, pragmatic study design based on prospective registry data with high external validity, use of patient-reported outcome measures, and protocol-based statistical analyses with blinded assessment of main outcome measures. The main limitation was the lack of randomization. Even though propensity-matched patient groups adjusts for known interactions, while unlikely, residual or introduction of confounding cannot be ruled out. Another weakness was the loss to follow-up of 30.6% of participants regarding Oswestry disability index scores at 1 year. A previous study on a similar population from the NORspine registry showed no difference in outcomes between non-responders and responders [21]. The minor differences in baseline characteristics between non-responders and responders at 1 year are not likely to influence our results [7, 13–15]. Also, we are lacking data on exact amounts of costs, payment, and reimbursements, inhibiting us from performing cost-effectiveness analyses.

## Conclusion

At 1 year, the effectiveness of microdiscectomy for lumbar disc herniation was equivalent for patients operated in private compared to public hospitals. However, patients operated in private clinics were managed more efficiently. Favorable outcomes were observed at 1 year in both treatment groups.
